# A missense mutation in the *suc22* gene encoding the small subunit of ribonucleotide reductase significantly sensitizes fission yeast to chronic treatment with hydroxyurea.

**DOI:** 10.17912/micropub.biology.001041

**Published:** 2023-12-19

**Authors:** Kajal Davi, Ilknur Yurtsever, Yong-jie Xu

**Affiliations:** 1 Pharmacology and Toxicology, Wright State University, Dayton, Ohio, United States

## Abstract

Ribonucleotide reductase (RNR) is essential for the biosynthesis of dNTPs and a therapeutic target. We have identified a missense mutation in *suc22* , which encodes the small subunit of RNR in fission yeast. The *suc22-S239F* mutation significantly sensitizes the cells to chronic but not acute treatment with the RNR inhibitor hydroxyurea. Preliminary data indicate that the drug sensitivity is likely due to decreased RNR activity. Since *S239F* is the first missense mutation reported for *suc22* and the mutated residue is highly conserved, the results will be useful for future yeast genetic studies and potentially, the development of new therapeutics targeting RNR.

**Figure 1. The suc22-S239F mutation significantly sensitizes the fission yeast S. pombe to chronic exposure, not acute treatment with HU:**
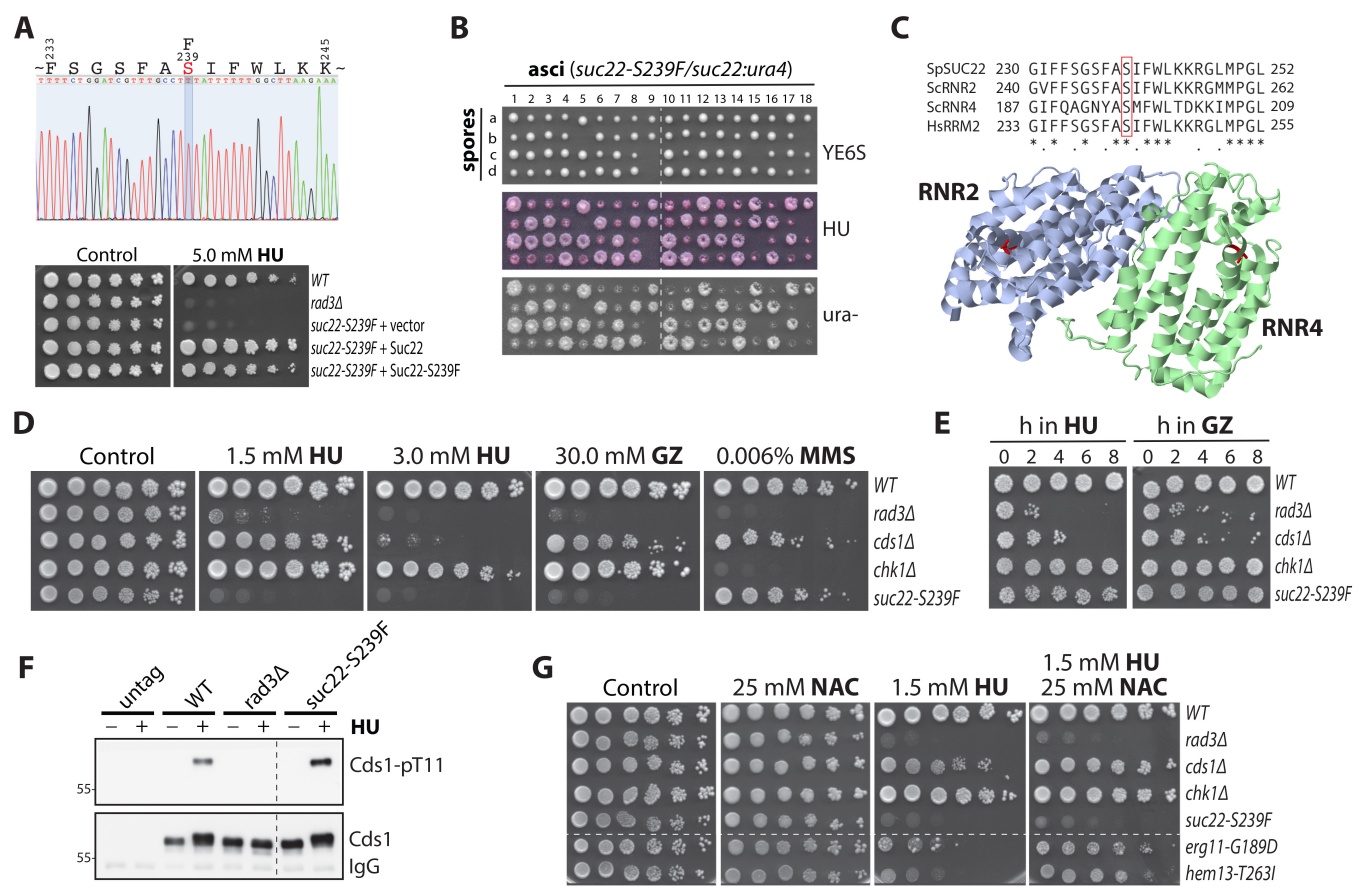
**(A)** Top panel: a genome-wide genetic screen uncovered a missense mutation in the *suc22* gene *.* DNA sequencing of the genomic locus identified a single C->T mutation in *suc22* , causing the S239F amino acid substitution. Lower panel: expression of wild-type and the mutant form of Suc22 from a vector under the control of its own promoter in *suc22-S239F* rescued the HU sensitivity. **(B)** Tetrad dissection of the asci from the genetic cross of the *suc22-S239F* mutant with a wild-type strain carrying *suc22* linked to the *ura4* marker. The colonies formed on the YE6S (yeast extract with six supplements) medium plate were replicated onto the medium plate containing 5 mM HU and phloxin B, a lethality indicator, or the plate lacking uracil. The dashed line indicates discontinuity. **(C)** The serine 239 residue in Suc22 is conserved from yeasts to humans. Top panel: ClustalW alignment of the regional amino acid sequences from *S. pombe* Suc22, the two RNR small subunits in the budding yeast *S. cerevisiae* RNR2 and RNR4, and the human RRM2. Asterisks and dots indicate absolute and high conservations, respectively. The mutated serine is highlighted in the red square. Lower panel: the conserved serine residues are highlighted in red in the crystal structure of the heterodimer of budding yeast RNR2 (purple) and RNR4 (green). **(D)** The sensitivity of the *suc22-S239F* mutant to HU, another RNR inhibitor guanazole (GZ), and the DNA damaging agent methyl methanesulfonate (MMS) were examined by spot assay. **(E)** Insensitivity of the *suc22-S239F* mutant to acute treatment with HU and GZ. Wild-type and mutant *S. pombe* with the indicated mutations were incubated with 15 mM HU or 100 mM GZ in YE6S liquid cultures. An aliquot of the culture was removed every 2 h during the incubation. The cells were washed and then spotted on YE6S plates to allow recovery. **(F)** The DNA replication checkpoint remains functional in the *suc22-S239F* mutant. *S. pombe* expressing Cds1 ^CHK2^ tagged with or without the HA epitope were treated with (+) or without (-) 15 mM HU for 3 h. Cds1 was immunopurified for Western blotting with an anti-HA antibody (lower panel). The blot was stripped and then probed with a phospho-specific antibody to reveal the phosphorylated threonine 11 in Cds1 ^CHK2^ by Rad3 ^ATR^ (top panel). The dashed line indicates discontinuity. **(G)** The HU sensitivity of *suc22-S239F* is unlikely due to oxidative stress. The HU sensitivity of wild-type *S. pombe* and cells with the indicated mutations were examined by spot assay in the presence or absence of 25 mM N-acetylcysteine (NAC), an antioxidant as in D. The dashed line indicates discontinuity.

## Description

Ribonucleotide reductase (RNR) catalyses the rate-limiting step of the biosynthesis of dNTPs (Jordan & Reichard, 1998) , the building block of DNA. Since adequate and balanced dNTP pools are essential for faithful DNA replication and repair, alterations of the dNTP pools cause genome instability and have been linked to multiple human diseases, including cancer (Aye et al., 2014) . Inhibitors of RNR, such as hydroxyurea (HU), gemcitabine, and clofarabine have been used clinically or are being developed for the treatment of cancer or other diseases (Gridelli et al., 2007; Narasimhan et al., 2022; Saito et al., 2021; Singh & Xu, 2016; Xu et al., 2006) . RNR inhibitors such as HU have also been widely used in laboratories for generating DNA replication stress or arresting cells in the S phase of the cell cycle (Singh & Xu, 2016) . Consistent with the mechanism of RNR inhibition, the primary response to HU treatment in proliferating cells involves the activation of the DNA replication checkpoint (DRC) in all eukaryotic organisms (Elledge et al., 1992) . The activated DRC mobilizes cellular pathways to mitigate the replication stress, protect the genome integrity, and promote cell survival (Boddy & Russell, 1999) . Since many anticancer drugs interfere with DNA replication, causing replication stress, and the DRC status of cancer can significantly influence the chemotherapeutic efficacies, RNR and the DRC have been of intense research interest in the past decades. To better understand the DRC mechanisms, we have carried out a large-scale genetic screening in the fission yeast *S. pombe* by random mutagenesis of the genome, looking for mutants that are sensitive to HU (Enoch et al., 1992) . This *hus* (HU sensitive) genetic screen has identified several DRC mutants as well as several metabolic mutants that are highly sensitive to HU (Ahamad et al., 2020; Khan et al., 2022; Singh & Xu, 2017; Xu et al., 2019; Xu et al., 2016) . Here, we report the identification and preliminary characterization of a previously unknown missense mutation in the *suc22* gene, which encodes the small subunit of RNR in fission yeast.

Preliminary data from the genetic crosses of this *suc22* mutant with the DRC mutants and other known *hus* mutants in fission yeast strongly suggest that it carries a previously unknown mutation. Extensive screening with *S. pombe* genomic libraries uncovered ≥ 10 plasmids that confer HU resistance to this mutant and all plasmids carried *suc22,* suggesting that the mutation is likely in the *suc22* gene. After sequencing the genomic locus, a single C->T mutation was identified in *suc22* , causing S239F amino acid substitution in the RNR small subunit ( Figure 1A, top panel ). To confirm the mutation, we expressed wild-type and the mutant Suc22 on a vector under the control of its own promoter in the *suc22* mutant. Spot assay showed that both the wild-type and the mutant Suc22 expressed on the vector rescued the mutant ( Figure 1A, lower panel ). However, the mutant Suc22 rescued the mutant to a lesser degree as compared to the wild-type protein, suggesting that *S239F* is a hypomorphic mutation, which is consistent with the essential function of *suc22* in *S. pombe* for cell growth. To provide further evidence, we linked *ura4* to the *suc22* gene at the genomic locus by integration in a wild-type strain. After crossing with the *suc22* mutant, both random spore analysis and tetrad dissection were performed on the resulting asci ( Figure 1B ). We found that the *hus* phenotype is segregated from the *suc22* -linked *ura4* marker in all spores. Together, these results confirm that the *S239F* mutation in *suc22* causes the *hus* phenotype in *S. pombe* .

RNR contains two large and two small subunits (Kang et al., 2020; Lebrette et al., 2023) . While the large subunit contains the catalytic site and two different allosteric sites, the small subunit harbors a di-iron cofactor and tyrosyl radical essential for catalysis. Unlike *S. pombe* , which expresses only one small RNR subunit that forms a homodimer in RNR, there are two small subunits namely RNR2 and RNR4 in budding yeast and two small subunits RRM2 and RRM2B in humans that form homodimers or heterodimers in RNR. Amino acid sequence alignment by ClustalW showed that the mutated serine 239 residue is highly conserved in eukaryotes, including the budding yeast RNR2 and RNR4 and the human RRM2 ( Figure 1C, top panel ). The crystal structure of the heterodimer of budding yeast RNR2 and RNR4 was solved over twenty years ago (Voegtli et al., 2001) . Like the bacterial RNR small subunit, both RNR2 and RNR4 are completely α-helical, and the conserved serine residue is within the αE helices of the two subunits ( Figure 1C, lower panel ).

HU inhibits RNR by quenching the tyrosyl radical in the small subunit (Lebrette et al., 2023; Nyholm et al., 1993) . Substitution of the serine 239 with the hydrophobic phenylalanine may perturb its structure locally, which may increase the accessibility of HU to the tyrosyl radical or affect the RNR activity, leading to the *hus* phenotype. To investigate these possibilities, we examined the sensitivities of *suc22-S239F* mutant to HU, another RNR inhibitor guanazole (GZ), and the DNA damaging agent methyl methanesulfonate (MMS) by spot assay ( Figure 1D ). We found that while the wild-type cells are resistant to all tested drugs, the cells lacking the master checkpoint kinase Rad3 ^ATR^ of both the DRC and the DNA damage checkpoint (DDC) pathways, are sensitive to all tested drugs. Since Cds1 ^CHK2^ is the effector kinase of the DRC, the *cds1∆* cells are more sensitive to HU and GZ than MMS. The *chk1∆* cells lacking the effector kinase of the DDC pathway, on the other hand, are more sensitive to MMS than HU and GZ. The *suc22-S239F* mutant was found sensitive to HU and GZ, not MMS, under similar conditions ( Figure 1D ), consistent with the mutation in RNR. The HU sensitivity of the mutant is remarkable in that it is even slightly more sensitive than *rad3∆,* which is one of the most HU-sensitive mutants known in *S. pombe* . We then examined the sensitivity of *suc22-S239F* to HU and GZ in liquid cultures ( Figure 1E ). While the DRC mutants *rad3∆* and *cds1∆* are highly sensitive to HU and GZ due to the lack of the DRC, the *suc22-S239F* mutant is, surprisingly, quite resistant to both HU and GZ during the 8 h long drug treatment. Consistent with the drug resistance observed in liquid culture, Rad3 ^ATR^ -dependent Cds1 ^CHK2^ phosphorylation in the DRC pathway remained unaffected or moderately increased ( Figure 1F ). This result shows that acute HU treatment in liquid culture generates replication stress at a normal or moderately increased level and the replication stress can be properly dealt with by the activated DRC in the mutant.

In addition to the replication stress, HU also generates oxidative stress, particularly under chronic drug exposure conditions like the spot assay shown in Figure 1D (Alyahya et al., 2022) . Since the mutation may destabilize the tyrosyl radical in Suc22, thereby generating reactive oxygen species, which may synergize with the HU-induced oxidative stress, causing the remarkable cell-killing effect as shown in Figure 1 D . To investigate this possibility, we examined whether the chronic HU sensitivity can be rescued by the antioxidant N-acetylcysteine (NAC) ( Figure 1G ). We found that while NAC can partially rescue our previously reported metabolic mutants *erg11-G189D* and *hem13-T263I* which are killed by HU mainly by oxidative stress (Singh & Xu, 2017; Xu et al., 2016) , it does not rescue the *suc22-S239F* mutant and the DRC mutants *rad3∆* and *cds1∆* . This result suggests that the remarkable chronic cell-killing effect of HU is likely due to replication stress, not oxidative stress in the *suc22-S239F* mutant. In support of this conclusion, the mutant is also sensitive to chronic treatment with GZ ( Figure 1D ) (Alyahya et al., 2022) , and a similar chronic sensitivity was observed when the cells were treated with HU under anaerobic conditions, where the endogenous oxidative stress is much reduced in cells. Although the exact cell-killing mechanisms of HU and GZ in *suc22-S239F* mutant remain to be fully understood, chronic exposure to the RNR inhibitors is likely sufficient to block the RNR-dependent dNTP synthesis and thus produce the remarkable cytotoxic effect. Since the *S239F* mutation is the first missense mutation reported for the essential gene *suc22* in fission yeast, the mutant should be useful for future genetic studies of unknown mutants with the *hus* phenotype. As the mutated serine residue is highly conserved in eukaryotes, the results reported here may also provide implications to the RNR studies in other model organisms and potentially the development of new therapeutics that target RNR.

## Methods

Yeast strains and the *hus* screen. The *S. pombe* strains were cultured at 30°C in YE6S medium (0.5% yeast extract, 3% dextrose, and 6 supplements) (Moreno et al., 1991) . Yeast strains used in this study are listed in the Reagents section. The method for screening for the *hus* mutants has been described previously (Singh & Xu, 2017; Xu et al., 2019; Xu et al., 2016) . The crystal structure of budding yeast RNR2 and RNR4 with the marked serine residues in Figure 1C is illustrated with POLYVIEW (Porollo, 2004) .

Drug sensitivity . The sensitivity of cells to HU, GZ, and MMS was determined by standard spot assay with a series of 5-fold dilutions as described in previous studies (Singh & Xu, 2017; Xu et al., 2016; Yue et al., 2011) .

Western analysis **.** Frozen cell pellets were lysed by mini-bead beater, and the HA-tagged Cds1 was immunopurified with anti-HA antibody beads from the lysates. Western blotting using anti-HA antibody and the phospho-specific antibodies against phosphorylated Cds1-Thr ^11^ was carried out as described in previous studies (Xu & Kelly, 2009; Yue et al., 2011) .

## Reagents

**Table:** 

**Strain**	**Genotype**	**Source**
TK48	* h ^-^ leu1-32 ade6-M216 *	Lab stock
NR1826	* h ^-^ rad3::ura4 leu1-32 ura4-D18 ade6-M210 *	Lab stock
GBY191	* h ^+^ cds1::ura4 leu1-32 ura4-D18 ade6-M216 *	Lab stock
TK197	* h ^+^ chk1::ura4 leu1-32 ura4-D18 ade6-M210 *	Lab stock
YJ1457	* h ^+^ suc22-S239F cds1-6his2HA(int) leu1-32 ura4-D18 ade6-M210 *	This study
YJ1451	* h ^-^ suc22:ura4 leu1-32 ura4-D18 ade6-M216 *	This study
YJ1296	* h ^+^ erg11-G189D:ura4 leu1-32 ura4-D18 ade6-M210 *	Lab stock
APS124	* h ^?^ hem13-T263I leu1-32 ura4-D18 *	Lab stock
